# Comment on 'Palovarotene reduces heterotopic ossification in juvenile FOP mice but exhibits pronounced skeletal toxicity'

**DOI:** 10.7554/eLife.43173

**Published:** 2019-01-30

**Authors:** Maurizio Pacifici, Eileen M Shore

**Affiliations:** 1 Translational Research Program in Pediatric OrthopaedicsThe Children's Hospital of PhiladelphiaPhiladelphiaUnited States; 2 Department of Orthopaedic SurgeryUniversity of PennsylvaniaPhiladelphiaUnited States; Maine Medical Center Research InstituteUnited States; University of OxfordUnited Kingdom

**Keywords:** rare skeleatal disorders, drug treatment, palovarotene, Mouse

## Abstract

We are writing to communicate our concerns regarding the recently published study by Lees-Shepard et al. (2018).

## Introduction

Lees-Shepard et al. reported that palovarotene (a retinoid agonist) had severe side effects on growth plate and limb joints in an Acvr1^R206H^ knock-in mouse model of fibrodysplasia ossificans progressiva (FOP; Lees-Shepard et al., 2018). We have a number of concerns about this publication. Lees-Shepard et al. write that “it is important for FOP therapeutics to exhibit an acceptable safety profile in juvenile patients”: however, they used a retinoid drug delivery method that is never used in the treatment of patients, thus undermining their goal. Moreover, the mouse model used does not sufficiently mimic FOP patients.

## Discussion

### Route of palovarotene administration

Palovarotene was originally designed for oral administration (Hind and Stinchcombe, 2009), allowing for low and steady drug adsorption by the digestive system and an overall absorption efficiency of 50% to 65% of administered dose, typical of retinoids (Blaner and Olson, 1994). Instead, Lees-Shepard et al. injected the drug intraperitoneally (IP) every day for 4 weeks. This IP route of administration is known to invariably cause a sharp and sudden increase in circulating drug levels, resulting in a higher and faster C_max_ compared to oral delivery (Eaton and Gilbert, 2013). Indeed, palovarotene C_max_ levels were found to be about 6-fold higher after IP injection compared to oral administration in mice (Clementia Pharmaceuticals, internal data). Circulating retinoids are quickly taken up by tissues and cells (Cullum and Zile, 1985), and cells accumulate retinoids in large amounts intracellularly (Kurlandsky et al., 1995; Randolph and Simon, 1997) where their action on cell function and phenotype can last hours to days (Chen and Gudas, 1996). Thus, daily IP injections are expected to result in ever increasing and possibly confounding drug effects over time. Lees-Shepard et al. stated that they used “palovarotene doses….that correspond to approximate adult human equivalent doses of 3.6 mg and 7.2 mg” under testing in ongoing efficacy and safety study of palovarotene for the treatment of FOP being conducted by Clementia Pharmaceuticals (https://clinicaltrials.gov/ct2/show/NCT03312634. This trial has recently entered phase 3). However, the IP administration changed the effective dose and undermined the goal of testing a clinically relevant treatment.

### FOP mouse model

Lees-Shepard et al. utilized a conditional mouse model in which the FOP causative gene was activated in Pdgfrα-expressing cells by mating floxed Acvr1^R206H^ mice with Pdgfrα-Cre mice. Those cells were described as a subpopulation of progenitor cells particularly sensitive to effects of the mutation. This mouse model is at sharp variance with FOP patients in which the mutant ACVR1^R206H^ gene characterizes the majority of, if not all, cells. Thus, data obtained with the Pdgfrα-Cre mouse model may be skewed by targeting a specific subpopulation of cells that could also be affected by wild-type cells, with the wild-type cells likely outnumbering the cells in the specific Acvr1^R206H^ subpopulation and interacting with them. Thus, the model does not faithfully reproduce the overall genetic makeup and cell and tissue responses in FOP patients. While Lees-Shepard et al. observed that Pdgfrα+ cells contributed to heterotopic ossification (HO) in their model, it is not known whether this specific cell population is clinically relevant in FOP patients. Further, Lees-Shepard et al. presented no data showing that targeted Pdgfrα+ cells were present in the growing skeleton.

### Effects on growth plates and joints

These latter issues are particularly relevant to the severe effects on growth plates and joints reported by Lees-Shepard et al. Given that the Pdgfrα gene is not known to be expressed in chondrocytes (Hamilton et al., 2003), it is nearly certain that growth plate and articular chondrocytes were not targeted by Pdgfrα-Cre and remained largely, if not totally, wild-type. Lees-Shepard et al. included no data to demonstrate that Pdgfrα-Cre had targeted growth plate and joint chondrocytes. Studies dating back decades have established that cartilage is sensitive to systemic retinoid levels (Wolbach and Hegsted, 1953). In our studies (Chakkalakal et al., 2016; Shimono et al., 2011), we did observe a slight growth retardation of about 10% in control wild type mice after oral palovarotene administration, although their growth plates remained open and there were no obvious effects on their joints. Notably, there were no obvious growth retardation and growth plate effects after oral palovarotene administration in our Prrx1-Cre; Acvr1^R206H^ mutant mice in which the entire limb mesenchymal cell population was targeted, including skeletal cells. The higher tolerance of mutant growth plates was likely due to higher basal levels of canonical BMP signaling activity. As we pointed out previously (Chakkalakal et al., 2016), this provides support to the notion that palovarotene may be quite safe for – and well tolerated by – FOP patients, as supported by the announcement of Phase 2 Part B data from the ongoing efficacy and safety study being conducted by Clementia (Clementia Pharmaceuticals, 2018).

### Conclusions

In sum, the severe side effects on growth plates and joints observed by Lees-Shepard et al. are likely the results of: (i) the route of drug administration; (ii) excessive palovarotene levels and effects likely caused by the daily IP administration; and (iii) reliance on a mouse model that does not mimic FOP patients, including apparent lack of mutation expression in cartilage. We strongly recommend that data and conclusions in that study should be considered and interpreted with caution with regard to drug effects and action in skeletal tissues and for relevance to ongoing clinical studies.
